# High-Dose Supplementation of Folic Acid in Infertile Men Improves IVF-ICSI Outcomes: A Randomized Controlled Trial (FOLFIV Trial)

**DOI:** 10.3390/jcm10091876

**Published:** 2021-04-26

**Authors:** Emmanuelle Mathieu d’Argent, Celia Ravel, Alexandra Rousseau, Karine Morcel, Nathalie Massin, Julie Sussfeld, Tabassome Simon, Jean-Marie Antoine, Jacqueline Mandelbaume, Emile Daraï, Kamila Kolanska

**Affiliations:** 1 Service de Gynécologie Obstétrique et Médecine de la Reproduction, AP-HP Sorbonne Université site Tenon, 4 rue de la Chine, 75020 Paris, France; julie.sussfeld@aphp.fr (J.S.); jean-marie.antoine@aphp.fr (J.-M.A.); emile.darai@aphp.fr (E.D.); kamila.kolanska@aphp.fr (K.K.); 2 CHU Rennes, Laboratoire de Biologie de la Reproduction-CECOS, Inserm, EHESP, Irset (Institut de Recherche en Santé, Environnement et Travail)-UMR_S 1085, Université de Rennes, 35000 Rennes, France; celia.ravel@chu-rennes.fr; 3 Department of Clinical Pharmacology and Clinical Research Platform of East of Paris (URCEST-CRC-CRB), APHP Sorbonne Universite, Hôpital Saint Antoine, 75012 Paris, France; alexandra.rousseau@aphp.fr (A.R.); tabassome.simon@aphp.fr (T.S.); 4 Centre d’Assistance Médicale à la Procréation, Service de Gynécologie-Obstétrique, CHU de Brest, Hôpital Morvan, 29609 Brest, France; karine.morcel@orange.fr; 5 Centre Hospitalier Intercommunal de Créteil, Department of Gynecology, Obstetrics and Reproductive Medicine, Université Paris-Est Créteil, 94010 Créteil, France; docmassin@gmail.com; 6 Service de Biologie de la Reproduction, Centre d’Etudes et de Conservation des œufs et du Sperme Humains (CECOS), Hôpital Tenon, Assistance Publique-Hôpitaux de Paris (APHP), Université Paris 06, 75020 Paris, France; mandelbaume.jacqueline@noos.fr

**Keywords:** folic acid, male infertility, sperm quality, IVF/ICSI outcome, antioxidants

## Abstract

Dietary supplementation is commonly used in men with male infertility but its exact role is poorly understood. The aim of this multicenter, randomized, double-blind, placebo-controlled trial was to evaluate the impact of high-dose folic acid supplementation on IVF-ICSI outcomes. 162 couples with male infertility and an indication for IVF-ICSI were included for one IVF-ICSI cycle. Male partners of couples wishing to conceive, aged 18–60 years old, with at least one abnormal spermatic criterion were randomized in a 1:1 ratio to receive daily supplements containing 15 mg of folic acid or a placebo for 3 months from Day 0 until semen collection for IVF-ICSI. Sperm parameters and DNA fragmentation before and after the treatment and the biochemical and clinical pregnancy rates after the fresh embryo transfer were analyzed. We observed an increase in the biochemical pregnancy rate and a trend for a higher clinical pregnancy rate in the folic acid group compared to placebo (44.1% versus 22.4%, *p* = 0.01 and 35.6% versus 20.4%, *p* = 0.082, respectively). Even if no changes in sperm characteristics were observed, a decrease in DNA fragmentation in the folic acid group was noted (8.5 ± 4.5 vs. 6.4 ± 4.6, *p* < 0.0001). High-dose folic acid supplementation in men requiring IVF-ICSI for male infertility improves IVF-ICSI outcomes.

## 1. Introduction

The prevalence of infertility is estimated at almost 15% in couples of reproductive age [1] and a male factor can be identified in around half of the cases [2]. Male infertility can have multiple origins such as genetic and environmental, or it can be associated with lifestyle [1]. However, in most cases the exact etiology remains unknown.

Some authors have reported that oxidative stress affects sperm parameters and live birth chances [3]. For example, in a review of 26 series on the combined use of antioxidants, Majzoub et al. reported a positive impact on semen parameters, advanced sperm function, outcomes of assisted reproductive therapy and live birth rates [4]. Similarly, in a review of 20 studies, Gharagozloo et al. showed a significant reduction in oxidative stress and improvement in sperm function parameters, as well as improved sperm deoxyribonucleic acid (DNA) fragmentation [5]. Finally, a recent Cochrane review of 48 randomized trials revealed that antioxidant therapy for male infertility improved pregnancy and live birth rates (odds ratio [OR] 4.21, 95% CI 2.08–8.51, *p* < 0.001) [6]. Consequently, dietary supplementation is commonly used for men in couples trying to conceive, but the exact role of the various antioxidant drugs is poorly understood.

**Figure 1:**
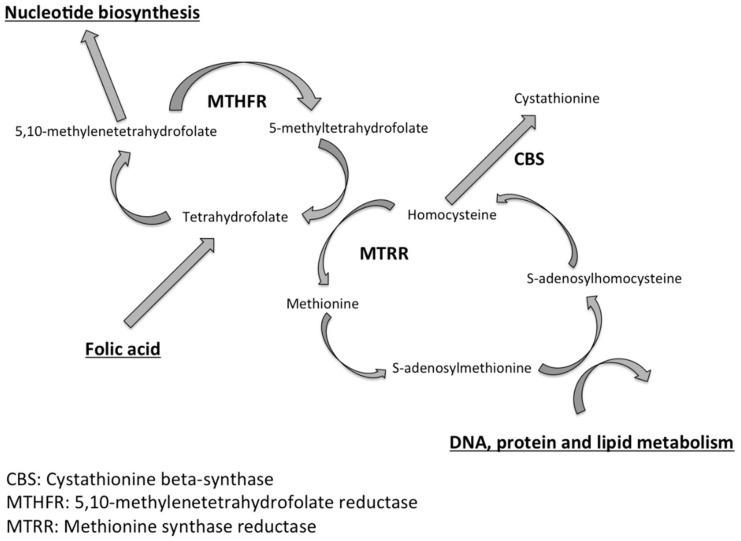
Key enzymes involved in the homocysteine and folate metabolic pathways.

The folate-related enzyme genes could be involved in male infertility [7,8]. Methylenetetrahydrofolate reductase (MTHFR) and methionine synthase reductase (MTRR) are key enzymes of the homocysteine and folate metabolic pathways [7] (Figure 1). Folate deficiency results in homocysteine overproduction with subsequent excessive oxidative stress [9], chaotic methylation reactions, protein synthesis, and spermatogenesis deficiency [10,11]. However, the exact role and recommended daily dose of folic acid necessary to improve spermatogenesis and spermatic quality parameters remain to be established. On one hand, previous studies have shown that administration of low doses of folic acid (5 or 10 mg per day) for a duration shorter than one cycle has no impact on spermatogenesis [12]. On the other hand, one study suggests an improvement in sperm quality and an increase in spontaneous pregnancies from the first cycle of spermatogenesis after folate supplementation for at least 3 months at a high dose of 15 mg per day [13].

Therefore, the aim of the present Randomized Controlled Trial (RCT) was to evaluate the impact of high doses of folic acid supplementation (15 mg per day for 3 months) compared to placebo on sperm characteristics and on in vitro fecundation—intracytoplasmic sperm injection (IVF-ICSI) outcomes.

## 2. Materials and Methods

### 2.1. Study Design

**Figure 2:**
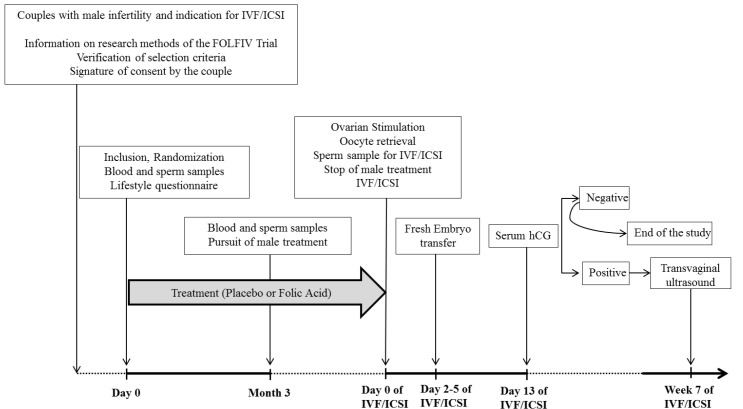
Study design. hCG—human chorionic gonadotrophin, IVF/ICSI—in vitro fecundation/intracytoplasmic sperm injection.

The FOLFIV trial was a multicenter, randomized, double-blind, placebo-controlled trial in couples with male infertility and an indication for IVF-ICSI. The aim was to evaluate the impact of folic acid supplementation on both sperm characteristics and IVF-ICSI outcomes. The trial protocol is described in Figure 2.

The study was approved by the French Health Products Safety Agency—ANSM and our Ethics Committee (Comité de Protection des Personnes, Ile de France XI, N° 11031, 12 May 2011), and registered on ClinicalTrials.gov (NCT01407432.) Written informed consent was obtained for all the participants.

### 2.2. Participants

We included male partners of infertile couples wishing to conceive, aged 18 to 60 years old, and requiring IVF-ICSI treatment for a male factor. Only men with at least one abnormal spermatic criterion according to the 2010 reference values of the World Health Organization (WHO) [14] were included in the study. Abnormal spermatic parameters were a sperm concentration <15 million spermatozoa per mL, motility <50% within 60 min of liquefication, and vitality <60% of live spermatozoa. Spermatozoa morphology was considered abnormal when <4% (or <5th centile) of the observed spermatozoa had normal morphology. Only couples with French National Health Insurance, who could speak and write French, with a female partner aged 18 to 38 years old, and without a female factor of infertility were included.

The exclusion criteria were the presence of a chronic viral disease (hepatitis B, hepatitis C, human immunodeficiency viruses) in either the male or female partner, a medical history of epilepsy, a poorly controlled chronic disease (for example, heart disease, diabetes, hypertension, cancer), prior cancer treatment, permanent obstructive disease of the deferent canal in the male partner, the need for testicular sperm or frozen sperm, supplementation with folic acid before the study, or medical intolerance to folic acid. Couples with a female partner presenting a known etiology of female infertility or ovarian failure defined as a blood follicle stimulating hormone (FSH) level >9 IU/L on day 3 of the menstrual cycle and/or antral follicle count (AFC) < 8 were also excluded from the study.

### 2.3. Protocol Procedures

On Day 0, after providing written consent, the male participants completed a questionnaire about their lifestyle, job, smoking history, and potential issues related to sexual intercourse. Sperm and blood samples were collected.

The men were then randomized in a 1:1 ratio to receive daily supplements either containing 15 mg (3 tablets of 5 mg each) of folic acid or a placebo (3 tablets a day) for 3 months as from Day 0. The study tablets were manufactured to match in appearance, size, taste and weight (Bailly-Creat Laboratory).

At the 3-month visit (M3), sperm and blood samples were collected and the sperm parameters were evaluated. Adverse event and adherence questionnaires were completed to assess relevant symptoms and the frequency of skipped doses. The treatment was continued until the semen collection for IVF-ICSI.

The IVF-ICSI procedure was planned 3–4 months after the beginning of the treatment. The women were monitored and managed according to institutional clinical protocols. Various controlled ovarian stimulation (COS) protocols were used: 150–450 IU/day of purified FSH or human menopausal gonadotropin in a gonadotropin-releasing hormone (GnRH) antagonist or long or short GnRH-agonist protocols. The gonadotropin dose and type of COS protocol were determined according to each patient’s characteristics (woman’s age, body mass index (BMI), AFC, anti-Mullerian hormone (AMH) serum level). Ovulation was triggered with choriogonadotropine alpha (Ovitrelle, Merck Serono^®^, Lyon, France) when at least three follicles had reached a diameter of 17 mm as assessed by transvaginal sonography. Transvaginal oocyte retrieval was scheduled 36 h after ovulation triggering and IVF/ICSI was performed with fresh sperm. The embryos were maintained in culture for 2 to 6 days and classified morphologically according to the Istanbul Consensus [15]. Fresh embryo transfer of one or two embryos was performed 1, 2–3 or 5 days after the oocyte retrieval. The luteal phase was supported by Utrogestan^®^ (Utrogestan 200 mg, Besins International, Montrouge, France) 400 mg vaginally twice per day starting on the day of the oocyte retrieval and continued until the 12th week of gestation (WG) when the patient was pregnant. Supernumerary embryos were cryopreserved on Day 2–3 or Day 5 according to their quality.

Each couple was enrolled for one IVF-ICSI cycle. Only the results of the fresh embryo transfer were taken into account for the primary endpoint. After embryo transfer, female participants were followed-up with a systematic blood sample for serum human chorionic gonadotrophin (hCG) on Day 13. If the test was positive, a transvaginal ultrasonography was planned for the 7th WG.

#### 2.3.1. Semen Analysis

Semen analysis was performed in either Tenon or Rennes University Hospital laboratories [14]. The sperm was collected in a sterile container by masturbation after 3–5 days of abstinence and analyzed after liquefaction according to the WHO criteria [14]. Parameters assessed were the ejaculate volume and sperm concentration, motility, vitality and morphology. The remaining semen samples were diluted in phosphate-buffered saline (PBS) and washed three times by centrifugation for 10 min at 700× *g*. Pellets were suspended in fixative (3:1 methanol/acetic acid) and frozen at −20 °C for further DNA fragmentation analysis.

#### 2.3.2. Evaluation of DNA Sperm Fragmentation by TUNEL Assay

All specimens for DNA fragmentation were addressed to the one central laboratory for analysis. Sperm DNA fragmentation was evaluated blindly by two biologists on 200 cells by fluorescence microscopy to quantify the percentage of spermatozoa incorporating deoxuridine triphosphate combined to fluorochrome in the breaks of DNA strands for enzymatic reaction divided by the total number of sperm nuclei labeled with 4’,6-diamidino-2-phenylindole (DAPI). DNA fragmentation was evaluated using the In Situ Cell Detection Kit (ref. 11684795910, Roche laboratory, Meylan, France^®^) according to the manufacturer’s instructions. Negative and positive controls, with and without DNase, respectively, were analyzed for validity testing. Sperm DNA fragmentation was evaluated with TUNEL assay using a terminal deoxynucleotidyl transferase-mediated 2´-deoxyuridine, 5´-triphosphate (dUTP) nick-end labeling. Analysis of DNA sperm fragmentation was performed as previously described by Ravel et al. [16].

#### 2.3.3. Blood Samples

At baseline (Day 0) and at M3, folic acid serum levels were analyzed by immunoassay with revelation by chemiluminescence (Architect CI 8200 Abott Diagnostic). We defined normal values at between 3.5 and 20.5 ng/mL. Folate deficiency was defined by a concentration <3.5 ng/mL.

### 2.4. Outcome Measures

The primary endpoints were:

- The biochemical pregnancy rate was assessed by the serum hCG level after the embryo transfer and considered positive when >100 IU/mL;
- The clinical pregnancy rate was assessed by ultrasonography at the 7th WG and considered positive when a gestational sac with at least one fetus with positive heart activity was observed.

The secondary endpoints were:

- Variations in sperm characteristics evaluated by comparing samples on Day 0 and at M3: volume of ejaculate, sperm concentration, motility, vitality;
- Variation in sperm DNA fragmentation was assessed on Day 0 and at M3.

Additional secondary endpoints were: number of oocytes retrieved, number of embryos obtained, number of top quality embryos and number of transferred and frozen embryos.

### 2.5. Randomization

A web-based randomization system was used. The randomization list was generated in a 1:1 ratio and was block balanced and stratified by center.

### 2.6. Statistical Analysis

The sample size calculation was based on the objective of 50% improvement in the pregnancy rate estimated at 32% among patients eligible for our study without treatment. To achieve 80% power, considering a two-sided alpha of 5% and 10% of dropout, 368 couples were needed.

The study population was analyzed per treatment group according to the intention-to-treat principle. The qualitative variables were described by their frequencies and percentages, and the quantitative variables by their means and standard deviations or medians and interquartile ranges (IQRs) according to their distribution. The pregnancy rate was compared between groups with a Pearson Chi-square test. The sperm parameters were compared using Wilcoxon-Mann-Whitney non-parametric test. The number of men with a folic acid serum level >40 g/L at M3 was compared using Fisher’s exact test. All tests were two-sided and *p* values < 0.05 indicated statistical significance. SAS V.9.3 software (SAS Institute Inc., Cary, NC, USA) was used for statistical analyses.

## 3. Results

**Figure 3:**
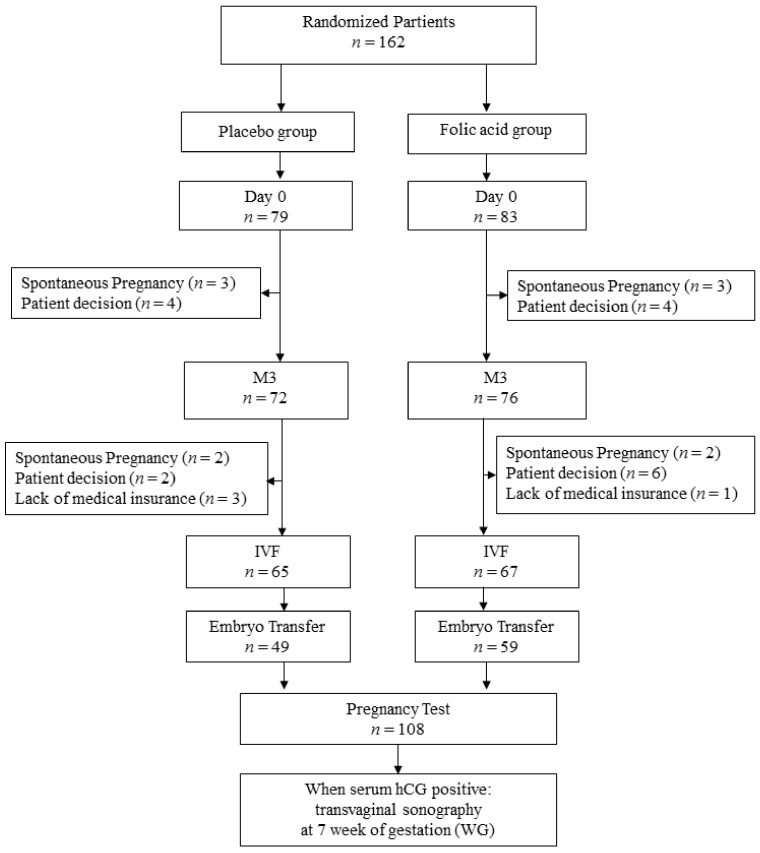
Flow chart. hCG—human chorionic gonadotrophin; IVF—in vitro fecundation; M3—month 3.

Participants were enrolled between November 2011 and September 2015 in six participating centers: Tenon Hospital, Rennes University Hospital, Intercommunal Center of Creteil Hospital, Pitié-Salpétrière Hospital, Monsouris Mutualist Institute, and Jean Verdier Hospital. The study population was composed of 162 couples randomized in the folic acid (*n* = 83) and placebo (*n* = 79) groups (Figure 3). Clinical data were completed in April 2016.

### 3.1. Epidemiologic Characteristics of the Population

IVF-ICSI procedures were done in 132 of the 162 couples (67 in the folic acid group and 65 in the placebo group). The reasons for exclusion of the 14 couples before IVF-ICSI are reported in Figure 3.

For the males, age, BMI, ethnicity, prior medical history, genital infection, or testicular trauma were similar in both groups (Table 1). None of the men included in the study were on other hormonal or vitamin treatments having a potential impact on fertility. No differences in prior testicular surgery including varicocele treatment were found between the groups. Testicular ultrasonography performed before inclusion in the study showed a higher rate of varicocele grade 1 to 3 in the folic acid group as compared to the placebo group (33% versus 49%, respectively). FSH serum levels were similar in both groups (5.71 ± 6.76 IU/mL in the folic acid group and 6.16 ± 8.28 IU/mL in the placebo group).

For the female partners, age, BMI, or ethnicity were comparable in both groups (Table 2). Seven women in the folic acid group had a clinical history of medical disease (one patient with diabetes, one with chronic respiratory disease, one with epilepsy, one with hypothyroidism, one with diabetes, one had allergies, and another a psychiatric disorder) as compared to five women in the placebo group (three had endocrinologic disorders—hypothyroidism, autoimmune hyperthyroidism and prolactin-producing pituitary adenoma—and two had a coagulation disorder). For fertility history, only one had endometriosis (in the folic acid group) and nine had polycystic ovary syndrome (five in the folic acid group and four in the placebo group). Ovarian reserve was similar in both groups (FSH and AMH serum levels and AFC) (Table 2).

For the couples (Table 3), the rate of primary infertility, pure male infertility, and duration of infertility were similar between the groups. The rate of couples with prior IVF-ICSI in the folic acid and placebo groups were 27.9% and 26%, respectively.

### 3.2. Semen Characteristics and Folic Acid Serum Levels on Day 0, at M3 and on the Day of IVF-ICSI

The sperm characteristics in each group are presented in Table 4.

At baseline (Day 0), the percentage of DNA fragmentation was similar in the folic acid and placebo groups (8.4% ± 4.6 and 15.0% ± 6.5 of labeled spermatozoa respectively, *p* = NS) (Table 4).

The M3 sperm characteristics for the folic acid and placebo groups are reported in Table 4. No difference in sperm characteristics (sperm concentration, total motility, progressive motility and morphology) was found between the groups.

The M3 folic acid serum level was higher in the folic acid group: a folic acid serum level over 40 µg/mL was observed in 61 men in the folic acid group as compared to one in the placebo group (80.3% versus 1.4%, *p* < 0.0001).

On the day of the IVF-ICSI procedure, no difference in the total motile sperm count between the groups was found (3.01 × 10^6^ ± 5.60 × 10^6^ in the folic acid group versus 1.91 × 10^6^ ± 2.66 × 10^6^ in the placebo group, *p* = 0.40).

### 3.3. Comparison between Baseline and M3

Sperm characteristics (sperm concentration, total motility, progressive motility, morphology and DNA fragmentation) in the folic acid and placebo groups on Day 0 and at M3 are reported in Table 4. No difference in sperm characteristics was found between Day 0 and M3 in each group and between the groups. In the folic acid group, the percentage of DNA fragmentation was lower at M3 than on Day 0 (*p* < 0.001). In the placebo group, no difference in DNA fragmentation was noted between Day 0 and M3.

### 3.4. Protocols of Ovarian Stimulation for IVF-ICSI

The duration of treatment was similar in both groups, with a median (IQR) of 4 months (4.0–5.0) in the placebo group compared to 4 months (3.0–4.0) in the folic acid group (NS).

The characteristics of the IVF-ICSI attempts for the folic acid and placebo groups are reported in Table 5. No differences in the rank of IVF-ICSI attempts, the stimulation protocol (GnRH-agonist or GnRH-antagonist), the duration of ovarian stimulation or the gonadotropin doses were found between the groups. No differences in the number of retrieved oocytes or the total number of embryos were found between the groups.

In the folic acid group, fresh embryo transfers were not performed for seven patients because of an inadequate endometrium (two cases) and the absence of viable embryos (five cases). In the placebo group, fresh embryo transfers were not performed in 12 patients because of ovarian hyperstimulation syndrome (two cases), an inadequate endometrium (two cases), a high level of progesterone at the end of ovarian stimulation (three cases), and the absence of viable embryos (five cases). No differences in the number of fresh transfers, the number of transferred embryos, the day of transfer and the number of frozen embryos were found between the groups.

### 3.5. Fertility Outcomes

Pregnancy rates are reported in Table 6. For the intention-to-treat population (*n* = 162), the total number of pregnancies (spontaneous and by IVF-ICSI) was 26 in the folic acid group and 15 in the placebo group (31.33% versus 18.99%, *p* = 0.071). Five spontaneous pregnancies between randomization and the IVF-ICSI attempt were noted in each group.

After IVF/ICSI, the number of biochemical pregnancies (serum hCG > 100 IU/mL) in the folic acid and placebo groups was 26 and 11, respectively (*p* = 0.02). The biochemical pregnancy rate by oocyte retrieval was higher in the folic acid group as compared to the placebo group (38.8% versus 16.9%, *p* = 0.005). The biochemical pregnancy rate by embryo transfer was significantly higher in the folic acid group (44.1% versus 22.4% respectively, *p* = 0.018). A trend for a higher clinical pregnancy rate by embryo transfer was observed in the folic acid group without reaching significance (35.6% versus 20.4%, *p* = 0.082). The miscarriage rate was comparable between the study arms (2% versus 8.5%, *p* = 0.146).

## 4. Discussion

The present randomized placebo-controlled study supports the hypothesis that supplementation by high-dose folic acid alone in men with infertility is associated with a higher biochemical pregnancy rate and with a trend for a higher clinical pregnancy rate after IVF-ICSI. Except for a decrease in DNA fragmentation in the folic acid group, no changes in sperm characteristics were observed after 3 months of treatment.

In this trial, the biochemical pregnancy rate, defined as serum hCG > 100 IU/mL per embryo transfer and per oocyte retrieved, was significantly higher in the folic acid group after IVF-ICSI. No differences in ovarian stimulation protocols, gonadotropins doses, number of retrieved oocytes, number of meiosis II oocytes or the number of embryos transferred were noted between the groups. Only a trend in a higher clinical pregnancy rate in the folic acid group was observed, which was probably linked to insufficient study power. These data might suggest an improvement in the embryo quality that could be a result of better sperm quality. The effect of antioxidant therapy on IVF-ICSI outcomes has already been suggested. In a randomized double-blind, placebo-controlled trial, Tremellen et al. demonstrated that antioxidant therapy including folic acid at 0.5 mg/day did not impact the biochemical pregnancy rate after IVF-ICSI (63.9% versus 37.5%, *p* = 0.077) [17]. However, the clinical pregnancy rate per embryo transferred was higher in the treated group as compared to the placebo group (38.5% versus 16%, *p* = 0.046). The number of couples included in the study was small, limiting the interpretation of the results, and the daily dose of folic acid administered was low. Bentivoglio et al. suggested that folate supplementation enhances spontaneous pregnancy only after at least 3 months of treatment at 15 mg/day [13]. However, in our study, the spontaneous biochemical pregnancy rate in couples assigned for IVF-ICSI treatment was similar in both groups.

A recent Cochrane review of 48 randomized controlled trials examining the effect of antioxidant therapy in male infertility revealed that pregnancy and live-birth rates were reported in only seven and four trials, respectively [6]. A significant improvement in the clinical pregnancy rate (odds ratio [OR] 3.43, 95% CI 1.92–6.11, *p* < 0.001) and live birth rate (OR 4.21, 95% CI 2.08–8.51, *p* < 0.001) were demonstrated. Ross et al. recently analyzed 17 randomized trials, including a total of 1665 infertile men in whom oral antioxidants were compared to placebo or no treatment [18]. Among the seven trials evaluating the pregnancy rate, six demonstrated a positive impact after antioxidant therapy. However, in a recently published multicenter randomized clinical trial by Schisterman et al. including 2370 men who received 5 mg of folic acid and 30 mg of elemental zinc daily (*n* = 1185) or placebo (*n* = 1185) for 6 months, no significant difference in live birth rates was found [19]. Once again though, the dose of administered folic acid was low.

It is not possible to determine the exact mechanism of action of folic acid treatment on sperm quality from the previously published studies. From a biological point of view, the present trial demonstrated that apart from DNA fragmentation, no difference was noted in sperm parameters after folic acid supplementation. Our results are in accordance with those of Eskenazi et al., showing no relation between folate supplementation combined or not with zinc supplementation on sperm features, especially for low dosages (5–10 mg/day) and for durations shorter than one cycle of spermatogenesis [12]. However, two randomized trials using folic acid supplementation at 5 mg daily for 26 weeks reported a low but significant increase in sperm concentration (mean increase of 7.5–9 million/mL) and a mild improvement in sperm nuclear quality suggesting that, beyond the folic acid dose, the duration of treatment should be taken into account [20,21]. Moreover, our results on folic acid serum levels are in agreement with those of Aarabi et al. who reported that an increase in serum folate concentrations after supplementation had no impact on sperm parameters [22]. In the meta-analysis by Irani et al. including four studies evaluating the effect of folate supplementation alone (5 mg/day) on endocrine and sperm parameters, no improvement in spermatozoa morphology (standard difference in means (SMD) −0.286 IC95% = [−0.58; 0.008], *p* = 0.06) or motility (SMD −0.088 IC95% = [−0.473; 0.296], *p* = 0.65) was found [23]. Although no improvement in conventional sperm features was noted, we observed a significant decrease in DNA fragmentation known to be associated with fertility enhancement [24,25,26]. Our results are in keeping with those of Aitken et al. and Zini et al., showing a positive impact of folic acid and zinc supplementation on sperm DNA alterations [5,22]. In contrast to sperm characteristics that do not predict fertility [27,28], previous studies have demonstrated that sperm DNA damage subsequent to oxidative stress should be considered an advanced test of sperm function correlated to fertility outcomes [24,26]. These data are in line with the review by Gharagozloo et al., including 20 trials which showed a significant reduction in oxidative stress evaluated by sperm DNA damage after oral antioxidant treatment [5], but in contradiction with the recent study by Schisterman et al. who observed a statistically significant increase in DNA fragmentation with folic acid and zinc supplementation compared to a placebo group (29.7% versus 27.2%; mean difference 2.4% [95%CI, 0.5–4.4%]) [19].

It has recently been suggested that myoinositol (another antioxidant treatment) has an impact on Assisted Reproductive Technology (ART) outcomes [29,30], giving rise to a discussion on different antioxidant treatment combinations.

Our results show that DNA fragmentation is lower after treatment in only a subpopulation of the men which is probably linked to the pathogenesis of the male infertility. These results are in accordance with a previous meta-analysis showing that not all infertile men may benefit from folic acid supplementation [8]. Indeed, the authors reported that approximately 20% of infertile men were homozygous for the MTHFR gene (677TT polymorphism) vs. 10% in the control group. Regardless of the fertility status, Ravel et al. found that two polymorphisms in the MTHFR gene (677CT and 1298AC) affected male fertility [16]: for men with 677CC polymorphisms supplementation of folic acid and zinc sulfate increased the number of spermatozoa from 20 to 45 million/mL (*p* = 0.005), while for men with CT heterozygotes and homozygotes TT exhibited no improvement in sperm characteristics [21]. Similarly, Boxmeer et al. showed the existence of an inverse correlation between folate concentration in seminal plasma and the DNA fragmentation rate in fertile men (standard adjusted regression coefficient −0.36, *p* ≤ 0.05) [31]. However, this correlation was not found in the overall study population. This might suggest that folic acid supplementation should be personalized to improve male fertility.

It is worth noting that, although our study showed an impact of folic acid supplementation on the biochemical pregnancy rate, there was no impact on the miscarriage rate. However, this was not the primary endpoint of the present study.

Some limits of the present study deserve to be underlined. Firstly, the number of patients included in the study was smaller than the estimated sample size and the a posteriori power was lower than expected (49%). This limits the definitive conclusion of folic acid supplementation in men requiring IVF-ICSI. However, the positive impact of high-dose folic acid on the biochemical pregnancy rate can be confirmed, even if only a trend was observed in the clinical pregnancy rate. Secondly, among the six centers which were initially registered to participate in the trial, only two actually participated in the inclusion process, which could represent a potential bias. Thirdly, the basal sperm parameters of men included in the study were more severely altered than expected. This could be because men with slight sperm parameter alterations were less keen to participate in the study as ART treatment was postponed for the duration of the folic acid treatment. However, the sperm parameters in both study groups were comparable. Fourthly, the fresh embryo transfers concerned mainly J2–J3 embryos, thus decreasing the selection criteria of transferred embryos. However, this embryo transfer policy was homogenous during the whole study period in all recruiting centers. Finally, the live birth rates were not analyzed. This item was not considered in the study design because more than 300 couples per arm would be required to demonstrate a significant difference taking into consideration the incidence of miscarriage after IVF/ICSI treatments.

## 5. Conclusions

Despite the limits of the present randomized trial, our results support the hypothesis that folic acid supplementation results in a higher biochemical pregnancy rate and a trend towards a higher clinical pregnancy rate after IVF-ICSI treatment in couples with male infertility. Moreover, the absence of change in conventional sperm features underlines the need for additional personalised criteria to evaluate the sperm quality, such as sperm DNA fragmentation.

## Figures and Tables

**Table 1:** Epidemiological characteristics, personal and family history of males in the folic acid and placebo groups.

Characteristics of the Men	Placebo Group (*n* = 79)		Folic Acid Group (*n* = 83)		*p*
	*n* *		*n* *		
Age (year), mean (SD)	79	36.5 (±6.2)	83	37.1 (±6.7)	0.5128
BMI (kg/m^2^), median (IQR)	74	25.3 (22.8–27.7)	73	26.5 (23.4–28.7)	0.2902
Ethnicity	79		83		0.4989
North African, *n* (%)		18 (22.7)		29 (35)	
Black African, *n* (%)		7 (8.9)		10 (12.1)	
South American, *n* (%)		1 (1.3)		1 (1.2)	
Asian *n* (%)		3 (3.8)		2 (2.4)	
Caucasian *n* (%)		44 (55.7)		36 (43.4)	
Others *n* (%)		6 (7.5)		5 (6)	
Medical history					
Family cryptorchidism ^1^, *n* (%)	76	1 (1.3)	78	1 (1.3)	0.8593
Pregnancy with previous partners, *n* (%)	77	3 (3.9)	83	5 (6.0)	0.3792
Mumps orchitis, *n* (%)	74	0 (0)	81	1 (1.2)	0.2682
Genital infection, *n* (%)	74	2 (2.7)	81	3 (3.7)	0.5371
Cryptorchidism, *n* (%)	74	0	80	0	0.5861
Testicular trauma, *n* (%)	75	3 (4.0)	81	3 (3.7)	0.7412
Treated for varicocele, *n* (%)	77	4 (5.2)	82	7 (8.5)	0.6035
History of testicular surgery ^2^, *n* (%)	77	4 (5.2)	82	2 (2.4)	0.4858
FSH serum level (IU/mL), median (IQR)	42	4 (0.0–6.7)	49	4 (0.0–7.9)	0.8179

BMI – body mass index, FSH—follicle stimulating hormone, IQR—interquartile range, SD—standard deviation, IU – international unit. ^1^ family history of cryptorchidism in a parent of the 1st or 2nd degree. ^2^ history of testicular surgery comprising testicular ectopia (*n* = 2), testicular torsion (*n* = 2), inguinal hernia (*n* = 1), penile surgery before age of 1 year (*n* = 1). * number of subjects with available data

**Table 2:** Epidemiological characteristics, personal and family history of females in the folic acid and placebo groups.

Characteristics of Women	Placebo Group (*n* = 79)		Folic Acid Group (*n* = 83)		*p*
	*n* *		*n* *		
Age (years) Mean (SD)	79	31.7 ± 4.1	83	31.3 ± 4.5	0.5514
BMI (kg/m^2^) median (IQR)	78	23.3 (20.9–26.6)	83	24.1 (21.5–26.1)	0.7331
Ethnicity	78		83		0.3254
North African *n* (%)		19 (24)		26 (31.3)	
Black African *n* (%)		8 (10.3)		14 (16.9)	
South American *n* (%)		2 (2.6)		1 (1.2)	
Asian *n* (%)		5 (6.4)		1 (1.2)	
Caucasian *n* (%)		39 (50.0)		35 (42.2)	
Others *n* (%)		6 (7.7)		6 (7.2)	
Endometriosis *n* (%)	78	1 (1.3)	83	0	0.2363
Polycystic ovary syndrome *n* (%)	78	4 (5.1)	82	5 (6.1)	1
FSH serum level (IU/mL), median (IQR)	70	4.7 (0.0–6.3)	74	1.5 (0.0–6.8)	0.7594
AMH serum level (ng/mL) median (IQR)	73	3.5 (2.1–5.4)	73	3.8 (2.1–5.7)	0.7571
AFC median (IQR)	63	14.0 (12–17)	60	13 (9.5–17.5)	0.4115

AFC—antral follicular count, AMH—anti-Mullerian hormone, BMI – body mass index, FSH—follicle stimulating hormone, IQR—interquartile range, SD—standard deviation, IU – international unit. * number of subjects with available data

**Table 3:** Epidemiological characteristics of the couples in the folic acid and placebo groups.

Characteristics of Couples	Placebo Group (*n* = 79)		Folic Acid Group (*n* = 83)		*p*
	*n* *		*n* *		
Primary infertility, *n* (%)	78	53(67.9)	83	56 (67.5)	0.9468
Etiology of the infertility:	79		83		0.9226
Male Infertility, *n* (%)		61 (77.2)		65 (78.3)	
Mixed Infertility, *n* (%)		17 (21.5)		18 (21.7)	
Duration of infertility (years) median (IQR)	79	3.0 (2.0–5.0)	83	3.0 (2.0–5.0)	0.4947
Previous infertility treatment:					0.8900
Intra-uterine insemination, *n* (%)		3 (3.8)		5 (6)	
IVF, *n* (%)		2 (2.5)		3 (3.6)	
ICSI, *n* (%)		22 (27.9)		26 (31.3)	

ICSI—intracytoplasmic sperm injection, IQR—interquartile range, IVF—in vitro fecundation. * number of subjects with available data

**Table 4:** Characteristics of sperm parameters on Day 0 and at M3 in the folic acid and placebo groups.

	Placebo Group (*n* = 79)		Folic Acid Group (*n* = 83)		*p*-Value
	*n* *		*n* *		
Spermatozoa concentration (Millions/mL)					
Day 0, median (IQR) mean ± SD	79	7.0 (3.0–18.0) 12.0 ± 13.3	83	5.5 (2.0–28.0) 21.9 ± 50.0	0.9360
M3, median (IQR) mean ± SD	72	7.7 (2.4–17.0) 11.0 ± 10.1	76	7.6 (2.5–24.0) 21.9 ± 38.6	0.6349
M3-M0, median (IQR) mean ± SD	72	0.0 (−4–3.1) −1.2 ± 11.3	76	−0.1 (−4.1–5.2) −1.2 ± 25.7	0.8088
Total motility (a + b + c)					
Day 0, median (IQR) mean ± SD	78	40.0 (25.0–50.0) 38.0 ± 18.4	82	45.0 (25.0–50.0) 38.3 ± 19.3	0.7457
M3, median (IQR) mean ± SD	72	40.0 (25.0–50.0) 38.4 ± 19.7	76	40.0 (20.0–55.0) 39.1 ± 20.3	1
M3-M0, median (IQR) mean ± SD	71	0.0 (−10.0–10.0) 0.0 ± 17.6	75	0.0 (−10.0–8.0) 0.1 ± 12.5	0.8830
Progressive motility (a) %					
Day 0, median (IQR) mean ± SD	66	0.0 (0.0–5.0) 4.3 ± 6.7	72	0.0 (0.0–10.0) 5.8 ± 8.4	0.4390
M3, median (IQR) mean ± SD	62	0.0 (0.0–10.0) 5.8 ± 8.6	67	0.0 (0.0–10.0) 6.4 ± 8.5	0.4390
M3-M0, median (IQR) mean ± SD	60	0.0 (0.0–5) 0.8 ± 6.9	66	0.0 (0.0–2) 0.6 ± 7.5	0.7871
Spermatozoa Morphology, % normal					
Day 0, median (IQR) mean ± SD	74	5.0 (2.0–10.0) 9.0 ± 11.7	80	6.0 (1.0–14.5) 9.9 ± 11.5	0.7513
M3, median (IQR) mean ± SD	70	5.0 (3.0–14.0) 9.7 ± 11.1	72	6.0 (1.0–16.0) 10.5 ± 12.2	0.8605
M3–M0, median (IQR) mean ± SD	66	0.0 (−2–4) 0.9 ± 7.3	71	0.0 (−2–4) 1.7 ± 10.0	0.6212
DNA fragmentation index (%)					
Day 0, median (IQR) mean ± SD	53	7.0 (5.5–10.5) 15.0 ± 6.5	52	8.0 (5.5–10) 8.4 ± 4.6	0.6963
M3, median (IQR) mean ± SD	53	5.5 (4.0–10.0) 7.4 ± 4.6	51	5.0 (3.0–9.0) 6.5 ± 4.6	0.1838
M3–M0, median (IQR) mean ± SD	53	−1.0 (−2.5–1) −65 ± 3.8	51	−2.0 (−4.5–0) −2.1 ± 3.3	0.0488

DNA—deoxyribonucleic acid, IQR—interquartile range, M0 – month 0, M3 – month 3, SD—standard deviation. * number of subjects with available data.

**Table 5:** Fertility outcomes after IVF in the folic acid and placebo groups.

	Placebo Group	Folic Acid Group	*p*-Value
Number of patients, *n*	65	67	
Attempt rank, *n* (%)			0.1402
1	44 (67.69)	43 (64.8)	
2	5 (7.69)	8 (11.94)	
3	7 (10.77)	13 (19.0)	
4	9 (13.85)	3 (4.48)	
IVF, *n* (%)	1 (1.54)	6 (8.96)	0.1153
IVF + ICSI *n* (%)	64 (98.46)	61 (91.04)	
Stimulation protocol, *n* (%)			0.6679
Long Agonist	14 (21.54)	20 (29.85)	
Short Agonist	9 (13.85)	10 (14.93)	
Flare Up agonist	2 (3.08)	1 (1.49)	
Antagonist	40 (61.54)	36 (53.73)	
Gonadotropin doses (IU), mean (SD)	1947.88 (±867.62)	2103.52 (±1267.95)	0.9059
Ovarian stimulation time (day), mean (SD)	11.38 (±1.57)	10.98 (±2.46)	0.5206
No. of retrieved oocytes, mean (SD)	12.7 (±7.1)	11.0 (±6.8)	0.2226
No. M II oocytes, mean (SD)	8.59 (±5.8)	7.6 (±5.32)	0.3735
No. of embryos obtained (Day1), mean (SD)	5.07 (±3.3)	4.83 (±3.17)	0.7255
Fertilization rate, mean (SD)	65.8 ± 39.3	68.0 ± 33.7	0.7541
No. of transfers, *n* (%)	49 (80.3)	59 (89.4)	0.1524
Day of fresh transfer, *n* (%)			0.7942
D1	1 (2)	1 (1.7)	
D2–D3	45 (91.8)	56 (94)	
D5	1 (2)	1 (1.69)	
No. of transferred embryos, mean (SD)	1.65 (±0.52)	1.58 (±0.5)	0.4803
No. of frozen embryos, mean (SD)	1.98 (±2.16)	1.81 (±2.3)	0.1402

D – day, ICSI—intracytoplasmic sperm injection, IVF—in vitro fecundation, M II—meiosis II, SD—standard deviation. For categorial variables, *n* (%) is presented. For continuous variables, mean ± standard deviation (SD) is presented.

**Table 6:** Pregnancy rates in the folic acid and placebo groups.

Pregnancy Rate	Placebo Group	Folic Acid Group	*p*-Value
Biochemical pregnancy rate per oocyte retrieval, *n* (%) *	11 (16.9)	26 (38.8)	0.005
Biochemical pregnancy per embryo transfer, *n* (%) **	11 (22.4)	26 (44.1)	0.018
Clinical pregnancy (7WG) per embryo transfer, *n* (%) **	10 (20.4)	21 (35.6)	0.082
Miscarriage rate per embryo transfer, *n* (%) **	1 (2)	5 (8.5)	0.146

WG—weeks of gestation; * *n* = 65 for the placebo group and *n* = 67 for the folic acid group; ** *n* = 49 for the placebo group and *n* = 59 for the folic acid group. For categorial variables *n* (%) is presented.
